# Effects of Schroth 3D Exercise on Adolescent Idiopathic Scoliosis: A Systematic Review and Meta-Analysis

**DOI:** 10.3390/children11070806

**Published:** 2024-07-01

**Authors:** Chenting Chen, Jialu Xu, Haifeng Li

**Affiliations:** 1 Department of Rehabilitation, Children’s Hospital, Zhejiang University School of Medicine, Hangzhou 310003, China; chenct@zju.edu.cn (C.C.); dew2020@zju.edu.cn (J.X.); 2 National Clinical Medical Research Center of Child Health and Disease, Children’s Hospital, Zhejiang University School of Medicine, Hangzhou 310003, China; 3 National Children’s Regional Medical Center, Children’s Hospital, Zhejiang University School of Medicine, Hangzhou 310003, China

**Keywords:** adolescent idiopathic scoliosis, Schroth, exercise training, systematic review, meta-analysis

## Abstract

(1) Background: This meta-analysis aims to systematically assess the effect size of Schroth three-dimensional exercise training on adolescent idiopathic scoliosis, especially for Cobb angles, angles of trunk rotation, and quality of life. (2) Methods: Randomized controlled trials (RCTs) focused on the effect of Schroth exercise on patients with adolescent idiopathic scoliosis (AIS) were retrieved from six databases, including PubMed, Embase, Cochrane Library, Web of Science, CNKI, and Wanfang. All publications until July 2023 were searched. Two researchers screened and evaluated the literature. Review manager (RevMan 5.3) statistical software was used for meta-analyses, and subgroup analysis and sensitivity analysis of the literature with high heterogeneity were further conducted. (3) Results: In total, 14 studies were included, including 538 adolescent idiopathic scoliosis patients. Compared with conventional physical therapy, Schroth 3D exercise training is more effective at reducing the Cobb angle (WMD = −3.32, 95%CI [−4.15, −2.50], *p* < 0.001) and improving the trunk rotation angle (WMD = −2.24, 95%CI [−3.00, −1.48], *p* < 0.001), quality of life (SMD = 2.80, 95%CI [1.53, 4.06], *p* < 0.001), and WRVAS (WMD = −2.92, 95%CI [−3.25, −2.60], *p* < 0.001), as well as enhancing the strength of the lumbar extensor (SMD = 1.79, 95%CI [1.46, 2.12], *p* < 0.001). (4) Conclusion: Compared with traditional therapy, Schroth 3D exercises are more effective at decreasing the Cobb angle and ATR in adolescent idiopathic scoliosis, improving patients’ quality of life, as well as enhancing the strength of the lumbar extensor.

## 1. Introduction

Idiopathic scoliosis is a term that was first brought up by Hippocrates and Galen hundreds of years ago, and Kleinberg in 1950 first described it as a three-dimensional deformity of the spine [1]. It is a multifactorial condition, and its etiology remains unclear. The diagnostic criterion proposed by the Scoliosis Research Society (SRS) defines scoliosis as a lateral curvature of the spine exceeding 10° in the coronal plane [2]. Adolescent idiopathic scoliosis (AIS) is the most common subtype of idiopathic scoliosis. Surveys indicate a prevalence of 1.23% among primary and secondary school students in mainland China, with a rising trend over the years. Moreover, the prevalence is approximately 1.6 times higher in females compared to males [3]. Clinical manifestations of AIS include leg length discrepancy and uneven shoulder level, among other postural issues [4]. In severe cases, individuals may experience back pain, psychological disturbances, and rarely, respiratory difficulties [5]. In recent years, AIS has emerged as the third most prevalent health issue among adolescents, following obesity and myopia [6].

The treatment approach for idiopathic scoliosis depends on the degree of spinal curvature, skeletal maturity, and patient age [7]. Treatment methods are broadly categorized into conservative and surgical interventions. Surgical treatments such as posterior spinal fusion (PSF) [8] can be applied in patients with severe curvature when the curve remains even when growth ends; since this therapy will result in significant tissue trauma and postoperative pain [9], we need to delay or avoid surgery whenever possible. In mild to moderate cases, conventional therapies are most commonly used to slow or stabilize the progression of scoliosis. The latest 2016 SOSORT guidelines (the Society on Scoliosis Orthopedic and Rehabilitation Treatment, SOSORT) include exercise training and bracing as part of conservative treatment options [10]. However, studies have shown that prolonged brace wear can have psychological effects on adolescents, leading to feelings of inferiority and identity issues [11]. Over recent years, exercise therapy has attracted the attention of therapies and clients. Schroth corrective training is one of the most popular physiotherapeutic scoliosis-specific exercise methods [12], which has received good feedback in clinic application. While a significant amount of research has been conducted on Schroth three-dimensional exercise training, studies still suffer from small sample sizes, insufficient high-quality research, and high heterogeneity [13,14,15]. In light of these challenges, this study aims to systematically evaluate the efficacy of Schroth three-dimensional exercise training compared to conventional treatments in adolescents with idiopathic scoliosis by incorporating the latest randomized controlled trials through a meta-analysis. The objective is to provide objective evidence-based support for the application of this technique in adolescents with idiopathic scoliosis.

## 2. Materials and Methods

### 2.1. Study Registration

This research protocol has been registered on the INPLASY International Systematic Review Registry platform (https://inplasy.com/ (accessed on 1 December 2023)) with registration number INPLASY2023120006. All the information is the same as the information provided at registration. The Preferred Reporting Items for Systematic Reviews and Meta-analyses (PRISMA) was used in conducting this research [16].

### 2.2. Study Inclusion and Exclusion Criteria

#### 2.2.1. Inclusion Criteria

The inclusion of literature follows the PICOS principles, as outlined in Table 1.

#### 2.2.2. Exclusion Criteria

The criteria of exclusion for studies included the following: (1) reviews, case reports, and conference reports; (2) duplicate publications; (3) studies with incomplete original data; (4) non-Chinese or non-English literature; (5) full text not accessible.

### 2.3. Literature Search

The databases searched included PubMed, Embase, Cochrane Library, Web of Science, CNKI, and Wanfang Database. Only studies in Chinese or English with full-text availability were considered, following specific PICOS criteria for inclusion in the meta-analysis. The retrieval scope encompassed publicly available clinical studies, with the search period extending until July 2023. The search strategies were as follows:

#1 schroth

#2 scoliosis or scoliosis or spine malformation or adolescent idiopathic scoliosis

#3 #1 AND #2

Chinese search strategies:

#1 schroth + 施罗斯 + 施罗特

#2 脊柱侧弯 + 脊柱侧凸 + AIS

#3 #1 AND #2

### 2.4. Literature Review and Data Extraction

Two researchers independently extracted data, resolving disagreements through discussion or involving a third researcher if needed. EndNote software (X9) was used to manage the included literature. Initial screening involved reviewing titles and abstracts, with full-text reading for unclear relevance. Excluded articles were documented with reasons for exclusion and counts. Researchers extracted data from the included literature, including first author’s name, year of publication, mean participant age, pre-treatment Cobb angle, angle of trunk rotation (ATR), Risser sign, scoliosis type, sample size, intervention details, intervention duration, and outcome measures. Data were extracted based on study grouping criteria, with efforts made to obtain missing information by contacting authors via email. Literature lacking essential data was excluded from further analysis if information could not be obtained.

### 2.5. Literature Quality Assessment

Two researchers independently assessed selected RCTs using the Cochrane Risk of Bias Assessment Tool [17]. Any discrepancies were resolved through discussion with a third researcher. The assessment evaluated several characteristics including random sequence generation (selection bias), allocation concealment (selection bias), participant and personnel blinding (performance bias), incomplete outcome data (attrition bias), selective reporting (reporting bias), and other biases, rating each as “low risk”, “high risk”, or “unclear risk” of bias. Overall assessment grades were determined based on the number of low-risk items identified, as follows: Grade A (four or more low-risk items), Grade B (two or three low-risk items), and Grade C (one or no low-risk items). Literature graded as C was excluded from subsequent analyses. Assessment results were statistically analyzed and presented with charts and figures to illustrate the quality assessment process of the randomized controlled trials comprehensively.

### 2.6. Statistical Analysis

Statistical analysis was conducted using Review Manager (RevMan 5.3) software and included the following components. 1. Effect measure: For dichotomous outcome data, the risk ratio (RR) and the odds ratio (OR) can be used to measure effects; for continuous outcome data, the weight mean difference (WMD) can be used to measure the absolute difference between two groups; the standardized mean difference (SMD) can be used when all studies present the same outcome, but the effect is measured in various ways, like different scales. Effect model: Considering that different kinds of intervention and different characteristics of patients will cause a variation effect, we chose random effects to calculate the effect size in all groups. Effect size calculation: Continuous outcomes were analyzed using mean differences (MDs) with corresponding 95% confidence intervals (CIs) to indicate the effect magnitude, with statistical significance set at *p* < 0.05. Heterogeneity assessment: Heterogeneity was assessed using the χ^2^ test and I^2^ statistic. *p* > 0.1 and I^2^ ≤ 50% indicate low heterogeneity; *p* ≤ 0.1 and I^2^ > 50% indicate substantial heterogeneity. Subgroup and sensitivity analyses were performed to explore sources of heterogeneity. These methods facilitated a comprehensive analysis, considering effect size and heterogeneity, and allowed for the exploration of subgroup differences and sensitivity to ensure robust and reliable findings.

## 3. Literature Search Results

### 3.1. Study Selection and Characteristics

A total of 742 articles were retrieved from various databases (PubMed: 158, Embase: 206, Cochrane: 89, Web of Science (WOS): 139, Medline: 80, CNKI: 26, Wanfang: 44), with an additional 1 article included from the references of selected studies. After removing duplicate articles using EndNote, 386 unique articles remained.

**Figure 1:**
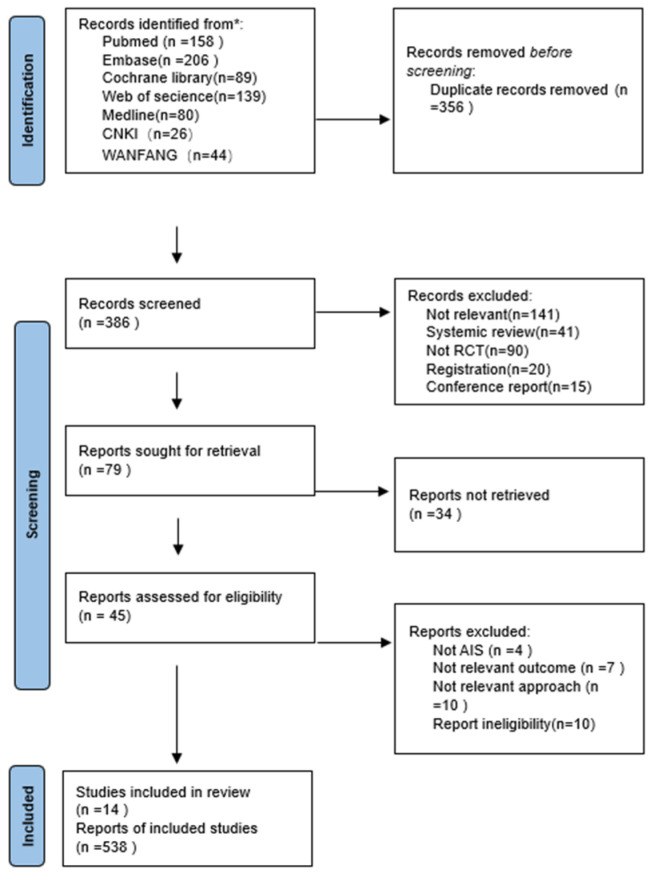
Flow chart search.

Following the screening of titles and abstracts, 79 articles were retained. Upon further examination of the full text, 34 articles were excluded due to the unavailability of the full text, leaving 45 articles. After a thorough full-text review, 27 articles were excluded for the following reasons: 4 did not focus on adolescents with idiopathic scoliosis, 7 had outcome measures that did not meet the inclusion criteria, 10 described interventions that did not match the criteria, and 10 had incomplete data. Ultimately, 14 articles were included in the study. The flowchart of the literature selection process is provided in Figure 1.

### 3.2. Basic Characteristics and Quality Evaluation

A total of 14 [18,19] articles were included in this study, all of which were RCTs involving 538 AIS. The basic characteristics of the included studies are summarized in Table 2.

Among these, 12 [19,20,21,22,23,24,25,26] articles comprising 508 AIS patients assessed changes in the Cobb angle before and after treatment. Seven articles [19,21,22,23,24,29,31] involving 295 AIS patients evaluated changes in the angle of trunk rotation (ATR) before and after treatment. Five articles [18,24,28,29,30] measured patients’ quality of life using the Scoliosis Research Society-22 (SRS-22) scale, with a total of 184 participants. Three articles [18,24,25] analyzed the self-image scores of patients, with one using the Spinal Appearance Questionnaire (SAQ) and two using the Walter Reed Visual Assessment Scale (WRVAS). Three articles [20,24,30] analyzed lumbar extensor strength, with one analyzing surface electromyography (EMG) and two analyzing back muscle strength. Only one article [27] evaluated patients’ lung function, assessing Functional Vital Capacity (FVC), Forced Expiratory Volume in one second (FEV1), FEV1/FVC ratio, and Maximum Voluntary Ventilation (MVV).

**Figure 2:**
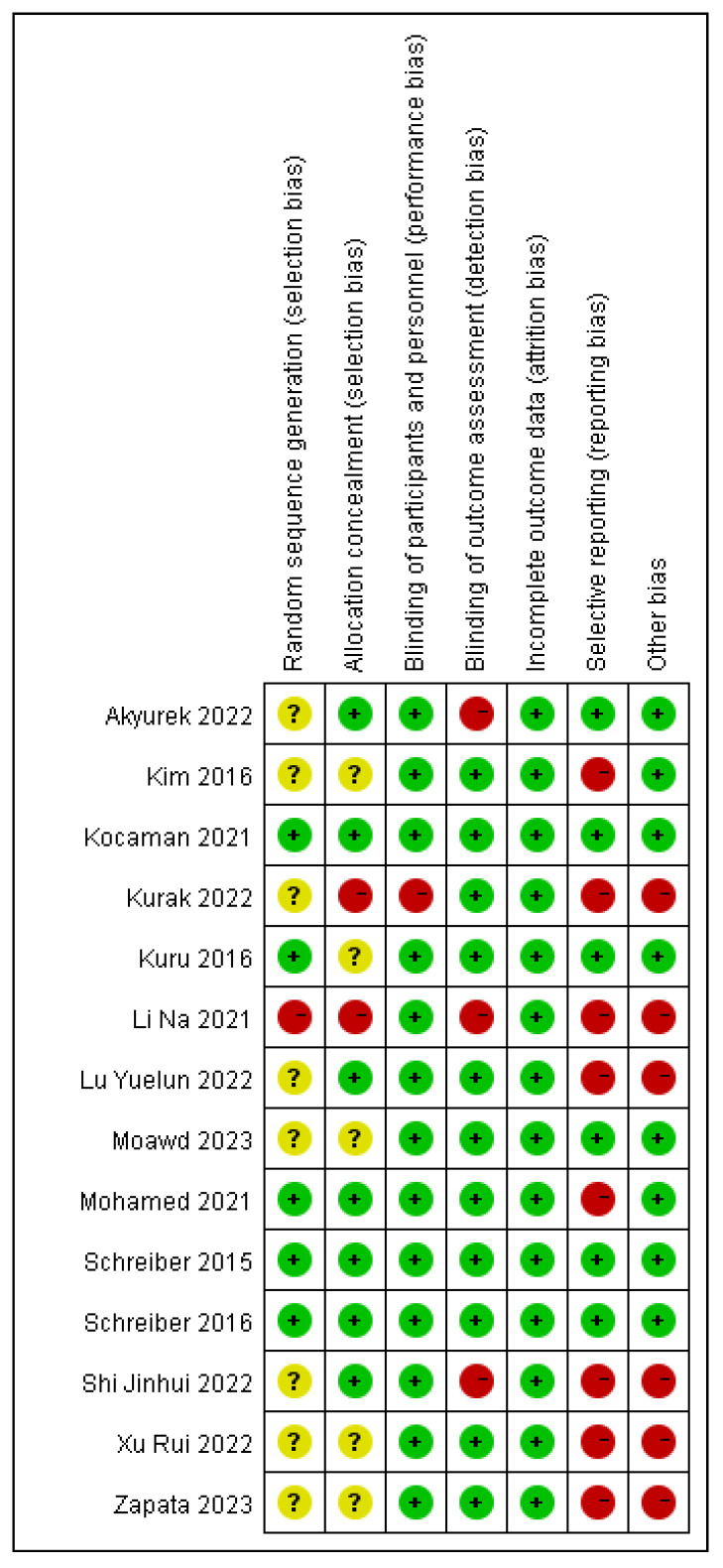
Risk of bias graph [18,19,20,22,24,25,26,27,28,29,30,31].

**Figure 3:**
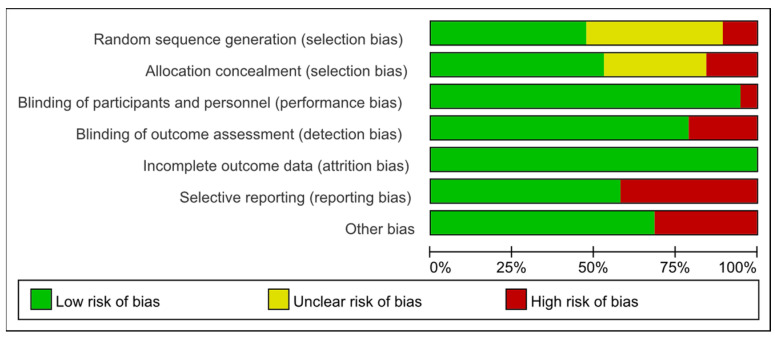
Summary of risk of bias.

The quality assessment of the included studies revealed nine articles of moderate quality and five articles of high quality. The risk of bias assessment for the included studies is presented in Figure 2 and Figure 3.

### 3.3. Result

#### 3.3.1. Cobb Angle

**Figure 4:**
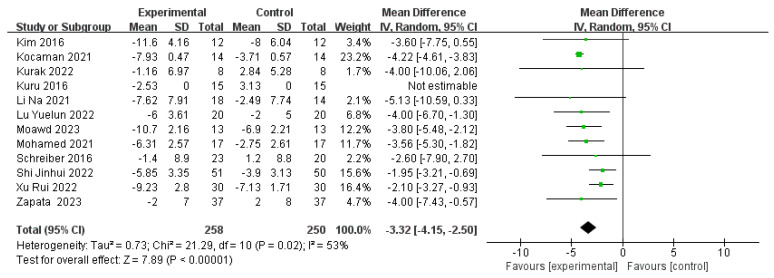
Effect size for the outcome of the Cobb angle. Refs. [19,20,22,24,26,27,28,29,30,31].

Twelve articles [19,20,21,22,23,24,25,26], involving 512 cases of adolescent idiopathic scoliosis (AIS), were analyzed using a random-effects model (*p* = 0.02, I^2^ = 53%). The results revealed a significant difference in Cobb angle scores between the experimental and control groups (WMD = −3.32, 95% CI [−4.15, −2.50], *p* < 0.00001), as depicted in Figure 4. Specifically, the Schroth training group demonstrated greater effectiveness in reducing the Cobb angle compared to the traditional treatment group. Given the observed heterogeneity among studies, subgroup analyses were performed based on potential influencing factors such as the pre-treatment Cobb angle, Risser sign, treatment duration, and intensity (refer to Table 3) to further elucidate sources of heterogeneity.

Subgrouping by pre-treatment Cobb angle resulted in lower heterogeneity among groups. Specifically, interventions targeting Cobb angles ranging from 10° to 20° demonstrated the highest effect size (WMD = −3.35, 95% CI [−4.11, −2.59]). Subgrouping based on Risser sign showed no significant heterogeneity among groups (I^2^ = 0%). Similarly, subgrouping by treatment intensity (daily vs. less than daily) also showed no significant heterogeneity among groups (I^2^ = 24% for daily, I^2^ = 41% for less than daily), suggesting that differences in pre-treatment Cobb angle, Risser sign, and treatment intensity contribute to the observed heterogeneity.

Additionally, subgrouping by total treatment duration revealed that interventions with a duration of 8–16 weeks had a higher effect size (WMD = −4.01) but exhibited significant heterogeneity (I^2^ = 74%). In contrast, interventions exceeding 16 weeks showed a lower effect size (WMD = −3.03) with no significant heterogeneity (I^2^ = 0%), suggesting that treatment duration alone is not the primary source of heterogeneity.

#### 3.3.2. ATR

**Figure 5:**
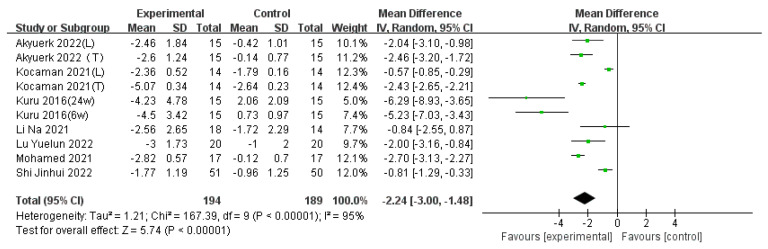
Effect size for the outcome of ATR. Refs. [19,22,24,25,29,31].

Seven articles [19,22,23,24,25,29,31] contributing 10 sets of data, involving a total of 295 cases of adolescent idiopathic scoliosis (AIS), were analyzed using a random-effects model (*p* < 0.00001, I^2^ = 95%). The analysis revealed that the experimental group had significantly lower ATR compared to the control group (WMD = −2.24, 95% CI [−3.00, −1.48], *p* < 0.01), as depicted in Figure 5. Due to substantial heterogeneity among studies, subgroup analyses were conducted to explore the sources of heterogeneity (I^2^ > 50%).

Subgrouping was performed based on pre-treatment Cobb angle, pre-treatment ATR angle, Risser sign, treatment duration, and treatment intensity. The results showed significant heterogeneity across all subgroups when stratified by pre-treatment Cobb angle, ATR angle, and Risser sign, indicating that these variables were not sources of heterogeneity. Subgrouping by treatment intensity revealed two groups with interventions administered 2 days per week and eight groups with interventions administered 3 days per week. The subgroup with interventions 2 days per week demonstrated a higher effect size (WMD = −2.32) with low heterogeneity (I^2^ = 0%), whereas the subgroup with interventions 3 days per week exhibited significant heterogeneity (I^2^ = 96%). Additionally, subgrouping by treatment duration showed significant heterogeneity across all subgroups, indicating that these factors were also not the primary sources of heterogeneity. The sources of heterogeneity may be related to publication bias, limited sample size, and type of scoliosis. Refer to Table 4 for details.

#### 3.3.3. SRS-22

Five articles [18,22,24,28,30] were included, comprising 199 cases of AIS, which were analyzed using a random-effects model. These five articles contributed data from 6 sets, with Kuru et al. [22] providing 22 sets of data for treatment durations of 6 weeks and 24 weeks. Among these articles, four utilized SRS-22 as the outcome measure, while one article used the SRS-23 version for assessment. Due to the use of different outcome measures, standardized mean difference (SMD) and 95% confidence intervals (CI) were employed as effect measures.

**Figure 6:**
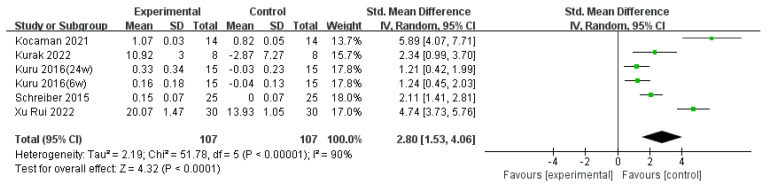
Effect size for the outcome of SRS-22. Refs. [18,22,24,28,30].

Heterogeneity testing of these six sets of data revealed significant heterogeneity (*p* < 0.00001, I^2^ = 90%), as depicted in Figure 6. Subgroup analyses were conducted based on pre-treatment Cobb angle, intervention intensity, and intervention duration to further explore the sources of heterogeneity. The results indicated that, except for the subgroup with an intervention duration exceeding 16 weeks, which did not demonstrate significant heterogeneity (I^2^ = 0%), all other subgroups showed significant heterogeneity. This suggests that these three subgroup variables were not the sources of heterogeneity. The sources of heterogeneity may be attributed to the limited number of included studies, individual differences, type of scoliosis, and publication bias. The reader can refer to Table 5 for details.

#### 3.3.4. WRVAS (Walter Reed Visual Assessment Scale)

**Figure 7:**
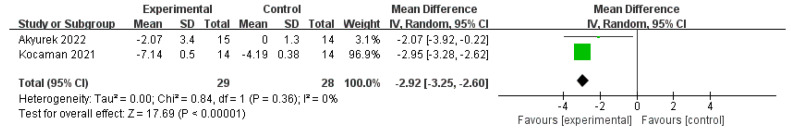
Effect size for the outcome of WRAVS. Refs. [24,25].

Two articles [24,25] were included, comprising 57 cases of AIS. The analysis indicated no significant heterogeneity among the studies (*p* = 0.42, I^2^ = 0%), allowing for the use of a fixed-effects model for the meta-analysis. The results revealed that Schroth exercise training led to greater improvement in WRVAS scores among AIS patients compared to the control group, with statistical significance (WMD = −2.92, 95% CI [−3.25, −2.60], *p* < 0.00001). This suggests that Schroth’s corrective therapy is more effective than traditional training in improving WRVAS scores for AIS patients. However, due to the limited sample size, the stability of the meta-analysis is relatively low, necessitating further discussion on the efficacy of improving patient self-image scores. The reader can refer to Figure 7 for details.

#### 3.3.5. Back Extensor Strength

**Figure 8:**
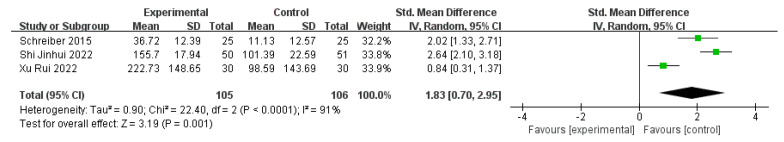
Effect size for the outcome of lumbar extensor strength. Refs. [18,19,30].

**Figure 9:**
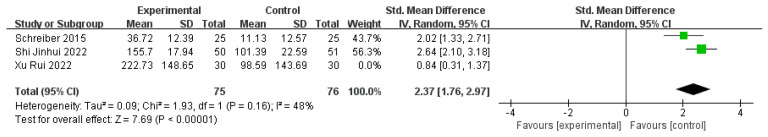
Leave-one-out analysis for the outcome of lumbar extensor strength. Refs. [18,19,30].

Three articles [18,19,30] were included, encompassing 211 cases of AIS. The analysis revealed significant heterogeneity among the studies (*p* < 0.00001, I^2^ = 91%). The results indicated that the experimental group in the included studies exhibited significantly higher lumbar extension strength compared to the control group (SWD = 1.83, 95% CI [0.7, 2.95], *p* < 0.0001). Substantial heterogeneity was observed among the three included studies when assessing the robustness through leave-one-out sensitivity analysis. Upon excluding the study by Xu Rui et al., heterogeneity decreased (*p* = 0.16, I^2^ = 48%). Upon careful examination of the articles, it was noted that Xu Rui et al. conducted treatment for only 6 weeks on patients, whereas the other two studies lasted 24 weeks. The difference in treatment duration may be a contributing factor to the observed heterogeneity. The reader can refer to Figure 8 and Figure 9 for details.

## 4. Discussion

The etiology of spinal curvature remains poorly understood [32]. Until now, the accepted view has been that the etiology can be divided into two parts, soft-tissue issues and bony issues. For soft tissue, the hypotheses are that the central nervous system corrects poor posture automatically in early stages of scoliosis; however, ongoing bilateral muscle imbalance causes the central nervous system to adapt to abnormal posture [33], ceasing corrective commands and ultimately leading to structural scoliosis. For bony tissue, skeletal spinal growth and bone metabolism can both effect spine mechanical strength and lead to the initiation and progression of spinal curve in AIS patients that occurs during the rapid growth period in pubertal growth spurts [34]. Thus, early prevention and correction are crucial in AIS. The Schroth method appeals to patients and therapists due to its personalized approach [35] and effectiveness. This study comprehensively assessed AIS treatment outcomes, using both objective (e.g., Cobb angle, ATR angle, lumbar strength, maximum expiratory volume) and subjective indicators (e.g., SRS-22, WRVAS scales) to evaluate changes in self-image and quality of life. The findings demonstrate that, in comparison to conventional therapy, Schroth exercise therapy exhibits superior efficacy in ameliorating patients’ conditions across various dimensions.

The Cobb angle and the ATR angle are the most commonly utilized indicators for assessing the severity of scoliosis [36]. This study shows that Schroth training significantly improves these angles compared to traditional methods, consistent with Park et al.’s findings [14]. Based on neuro-motor control theory, Schroth training targets spinal curvature and respiratory function through corrective exercises and respiratory training [37,38]. Therapists guide patients to extend the concave trunk area and reduce prominence using respiratory mechanics, activating weak muscles and relaxing tense ones to improve curvature angles. The subgroup analysis reveals that treatment outcomes vary based on the initial Cobb angle, Risser sign, and treatment intensity, emphasizing their importance in treatment planning. Treatment effectiveness is higher with durations of 8-16 weeks (effect size −4.01) versus longer durations (>16 weeks, effect size −3.03), and lower daily training intensity (effect size −2.66) is more effective due to reduced fatigue. However, given the limited sample size, potential bias exists, warranting further discussion on optimal training intensity for effective Schroth corrective therapy.

Spinal deformity impacts not only physiological function but also self-esteem and mental health [39]. Clinical treatment should prioritize patients’ self-image and psychological well-being [40]. This study used the WRVAS scale to track changes in self-image scores and the SRS-22 to assess quality of life. WRVAS is a reliable indicator of self-image in scoliosis patients, with higher scores indicating a greater perception of spinal deformity. The SRS-22 assesses functional status, pain, self-image, psychological status, and treatment satisfaction [41], with higher scores indicating greater treatment satisfaction. The meta-analysis results demonstrate that Schroth training significantly improves self-image and quality of life. Additionally, lumbar extensor strength improved significantly after Schroth training, likely due to posture correction, muscle length restoration, and activation of inhibited muscles [18].

This study also analyzed the impact of training methods on improving lung ventilation capacity, using FVC as the evaluation index [27]. Improved lung function is crucial for scoliosis patients considering surgery [42]. Schroth therapy significantly enhanced FVC, likely due to its emphasis on respiratory training, directing inhaled gas into the spinal curve’s concave area to activate muscles and improve respiratory function. However, very few studies have carried out a meta-analysis on this aspect, suggesting a need for further research into Schroth exercise therapy and lung function improvement.

## 5. Limitations

(1) The limited number of high-quality RCTs included diverse outcome indicators (back muscle strength, WRVAS, FVC), and variations in intervention type (some RCT interventions include Schroth exercise with other therapy, like massage and brace, while some only apply Schroth exercise), duration, frequency, and outcome measures could influence in heterogeneity. (2) This study focuses exclusively on adolescent idiopathic scoliosis (AIS), excluding juvenile idiopathic scoliosis and adult scoliosis and limits the generalizability of findings to other ages of scoliosis patients. (3) The scarcity of controlled trials in the literature categorizing types of scoliosis impedes the analysis of the effectiveness of Schroth corrective training for different curvature types.

## 6. Conclusions

In summary, Schroth exercise therapy shows notable benefits in treating idiopathic scoliosis by improving curvature and vertebral rotation angles, quality of life, and lumbar extensor strength compared to traditional treatments. However, rehabilitation outcomes vary due to multiple factors, highlighting the need for tailored treatment intensities based on individual patient needs. Standardized training programs guided by physical therapists are recommended for treatment customization and effectiveness. Future experimental studies should explore the relationship between Schroth corrective training and respiratory function improvement. 

## Figures and Tables

**Table 1:** PICOS Criteria for the inclusion of literature.

PICOS	Inclusion Criteria
Population (P)	Adolescents with idiopathic scoliosis, 10° < Cobb angle < 45°, Risser stage < V
Intervention (I)	Primary use of Schroth training as the main treatment method
Comparison (C)	Primary use of conservative treatment excluding Schroth training
Outcome (O)	Cobb angle, angle of trunk rotation (ATR), Scoliosis Research Society-22 (SRS-22) quality of life questionnaire, pulmonary function (FVC, FEV1, FEV1/FVC, MVV, and 6MWT), Walter Reed Visual Assessment Scale (WRVAS); lumbar extensor
Study Design (S)	Randomized controlled trials (RCTs)

**Table 2:** Description of the studies.

Study	Age		N		Subject Type				Program Type			Intensity	Outcome
	E	C	E	C	Type	Risser Sign	Cobb Angle	ATR	E	C			
Schreiber 2015 [18] Schreiber 2016 [20]	(13.4 ± 1.6) /(13.4 ± 1.6)		25/25		3c (n = 7), 3cp (n = 15), 4cp (n = 23), 4c (n = 5)	1.76/1.44	29.1° /27.9°	Not available	Schroth	Observation or bracing		1 h/d, qd, 3 m and 6 m	Biering-Sorensen (BME) test, Scoliosis Research Society (SRS-22r), Spinal Appearance Questionnaire (SAQ) scores, largest Cobb, sum of curve
Kim 2016 [21]	(15.6 ± 1.1) /(15.3 ± 0.8)		12/12		Not available	Not available	24.0 ± 2.6° /23.63 ± 1.5°	Not available	Schroth	Pilates		60 min, 3 d/w, 12 w	Cobb, weight distribution
Kuru 2016 [22]	(12.9 ± 1.4) /(13.1 ± 1.7) /(12.8 ± 1.2)		15/15/15		Not available	1.5 ± 1.3 /1.4 ± 1.4 /1.0 ± 1.2	33.4 ± 8.9° /30.3± 7.6° /30.3 ± 6.6°	11.9 ± 5.2°	Schroth	Schroth home exercise	Control	1.5 h, 3 d/w, 24 w	Cobb, ATR, waist asymmetry, maximum hump height, srs-23
Mohamed 2021 [23]	(14.50 ± 1.2) /(14.9 ± 1.4)		17/17		Not available	3/3.11	20.42 ± 2.57° /20.21 ± 2.80°	8.05 ± 0.65° /8.29 ± 0.68°	Schroth	PNF		3 d/w, 24 w	Cobb, ATR, static plantar pressure, 6MWT
Kocaman 2021 [24]	(14.07 ± 2.37) /(14.21 ± 2.19)		14/14		Lenke I, RT = 3, LT = 5, RTLL = 6 vs. Lenke I, RT = 3, LT = 5, RTLL = 6	1.64 ± 1.34	10°–20°	ATR-T (8.71 ± 2.37°) ATR-L (4.29 ± 2.73°)	Schroth	Core		90 min, 3 d/w, 10 w	Cobb, ATR, WRVAS, spinal mobility, peripheral muscle strength, srs-22
Akyurek 2022 [25]	(13.73 ± 1.83) /(13.86 ± 1.86)		15/14		TriLle/TleLri = 7, LriTle/LleTri = 7 TriHle = 1 vs. TriLle/TleLri = 7, LriTle/LleTri = 7. TriHle = 0	2.50 ± 1.65 /2.71 ± 2.05	<25°	ATR-T (5.47 ± 4.29° /6.29 ± 3.75°) ATR-L (5.53 ± 3.09° /6.00 ± 3.53°)	Schroth	Schroth home exercise		2 d/w, 8 w	JR error, ATR, posture parameters, WRVAS
Zapata 2023 [26]	(12.7 ± 1.3) /(12.1 ± 1.0)		37/37		L = 22, TL = 15 vs. L = 23, TL = 14	After 1 year: 1.6 ± 1.4 /2.3 ± 1.7	20°–30°	Not available	Schroth + nighttime bracing	Nighttime bracing		15 min/d, 5 d/w, 1 y	Cobb, rate of cure progression, surgery recommended
Moawd 2023 [27]	(13.5 ± 1.2) /(13.8 ± 1.5) /(14.1 ± 1.2)		13/13/12		Not available	Not available	35°–40°	4.5 ± 1.4° /5.2 ± 1.3° /4.9 ± 1.3°	Schroth + brace	Brace	Conventional exercises	Qd, 8 w/24 w	Cobb, ATR, FVC, FEV1, FEV1/FVC, MVV
Kurak 2022 [28]	14–16		8/8/8		T	Not available	32.54 ± 5.80°/ 33.26 ± 8.05°/ 31.86 ± 5.12°	Not available	EMS Schroth	Schroth	Control	3 d/w, 26 w	Cobb, SRS-22
Li Na 2021 [29]	10–16		18/14		T = 9, L = 2, TL = 7 vs. T = 7, L = 2, TL = 5	≤IV	33.00 ± 8.13° /31.62 ± 8.15°	10.28 ± 2.88° /9.79 ± 2.35°	Schroth + brace	Brace		90 min/d, 3 d/w, 16 w	Cobb angle, PO, CA, TK, LL, ATR, vertebral angel and SRS-22
Xu Rui 2022 [30]	12.57 ± 1.31/12.37 ± 1.25		30/30		Not available	I-IV	21.70 ± 2.26° /21.30 ± 1.93°	Not available	Schroth	Massage		30 min/d, qd, 8 w	Cobb, SRS-22, trunk muscle strength and angle, clinic efficacy
Lu yuelun 2022 [31]	13.8 ± 2.1/13.7 ± 3.9		20/20		Not available	Not available	15 ± 5° /14 ± 4°	5 ± 2° /5 ± 2°	Massage + Schroth	Massage		90 min/ d, 3 d/w, 6 m	Cobb, ATR, clinic efficacy
Shi jinhui 2022 [19]	13.85 ± 1.54/14.09 ± 1.78		51/50		T = 12, L = 23, L = 16 vs. T = 10, L = 27, TL = 13	≤IV	30.74 ± 3.86° /31.32 ± 3.52°	5.47 ± 1.32° /5.59 ± 1.27°	Schroth + brace	Brace		1.5 h/d, 3 d/w, 24 w	Cobb, ATR, CA, ATR, trunk muscle strength

Note: TriLe/TleLr: thoracal major double curve; LriTle/LleTri: lumbar major double curve; TriHle: single curve; 3c: thoracic single curve with pelvic balance; 3cp: thoracic single curve with pelvic tilt; 4c: double curve with pelvic balance; 4cp: double curve or lumbar curve with pelvic tilt; PO: pelvic oblique; CA: clavicular angel; TK: thoracic kyphosis; LL: lumbar lordosis.

**Table 3:** Subgroup analysis for outcome of the Cobb angle.

Subgroup	Effect Size	*p* Value	I^2^%	P Heterogeneity	Z
**1.1 Cobb**					
1.1.1 10°–20°	−4.22 [−4.60, −3.83]	<0.00001	0%	0.87	21.57
1.1.2 20°–30°	−2.71 [−3.61, −1.81]	<0.00001	0%	0.61	5.89
1.1.3 >30°	−2.92 [−4.23, −1.61]	<0.0001	24%	0.27	4.36
**1.2 Risser Sign**					
1.2.1 I–II	−4.21 [−4.59, −3.83]	<0.0001	0%	0.083	21.51
1.2.2 II–III	NA	NA	NA	NA	NA
1.2.3 >III	−3.56 [−5.30, −1.82]	<0.0001	NA	NA	4.01
**1.3 Intensity**					
1.3.1 qd	−2.66 [−3.60, −1.71]	<0.0001	24%	0.27	4.41
1.3.2 <qd	−4.01 [−4.36, −3.65]	<0.0001	41%	0.11	7.67
**1.4 Duration**					
1.4.1 8 w–16 w	−4.01 [−4.38, −3.65]	<0.0001	74%	0.009	4.16
1.4.2 >16 w	−3.03 [−3.82, −2.24]	<0.0001	0%	0.56	7.5

NA: Not applicable.

**Table 4:** Subgroup analysis for outcome ATR.

Subgroup	Effect Size	*p* Value	I^2^%	P Heterogeneity	Z
**1.1 cobb**					
1.1.1 10°–20°	−1.88 [−2.94, −0.82]	0.0005	96%	<0.00001	3.49
1.1.2 20°–30°	−2.70 [−3.13, −2.27]	<0.00001	Not applicable	Not applicable	12.33
1.1.3 >30°	−3.12 [−5.69, −0.54]	0.02	92%	<0.00001	2.37
**1.2 ATR**					
1.2.1 <5°	−1.17 [−2.55, 0.21]	0.1	82%	0.02	1.66
1.2.2 5°–10°	−2.09 [−2.78, −1.40]	<0.00001	91%	<0.00001	5.91
1.2.3 >10°	−4.03 [−7.42, −0.65]	0.02	88%	0.0002	2.33
**1.3 Risser Sign**					
1.3.1 I–II	−3.10 [−4.65, −1.55]	<0.00001	98%	<0.00001	3.92
1.3.2 II–III	−2.32 [−2.93, −1.72]	<0.00001	0%	0.52	7.51
1.3.3 >III	−2.70 [−3.13, −2.27]	<0.0001	Not applicable	Not applicable	12.33
**1.4 Intensity**					
1.4.1 2 d/w	−2.32 [−2.93, −1.72]	<0.00001	0%	0.52	7.51
1.4.2 3 d/w	−2.25 [−3.15, −1.36]	<0.0001	96%	<0.00001	4.91
**1.5 Duration**					
1.5.1 <8 w	−5.23 [−7.03, −3.43]	<0.00001	Not applicable	Not applicable	5.7
1.5.2 8 w–16 w	−1.71 [−2.80, −0.61]	0.002	96%	<0.00001	3.05
1.5.3 >16 w	−2.50 [−3.94, −1.07]	0.0006	93%	<0.00001	3.42

**Table 5:** Subgroup analysis for the outcome of SRS-22.

Subgroup	Effect Size	*p* Value	I^2^%	P Heterogeneity	Z
1.1 cobb					
1.1.1 10°–20°	5.89 [4.07, 7.71]	<0.00001	Not applicable	Not applicable	6.34
1.1.2 20°–30°	3.40 [0.82, 5.98]	0.01	94%	<0.0001	2.58
1.1.3 >30°	1.40 [0.85, 1.96]	<0.00001	11%	0.32	4.94
1.2 intensity					
1.2.1 <3 d/w	2.47 [0.94, 3.99]	<0.0001	87%	<0.0001	3.17
1.2.2 ≥3 d/w	3.40 [0.82, 5.98]	<0.0001	94%	<0.0001	3.27
1.3 duration					
1.3.1 <8 w	1.24 [0.45, 2.03]	0.002	Not applicable	Not applicable	3.07
1.3.2 8 w–16 w	3.87 [0.96, 6.79]	0.009	95%	<0.00001	2.61
1.3.3 >16 w	2.16 [1.54, 2.78]	<0.00001	0%	0.76	6.79

## References

[1] Kleiberg S. (1950). Sciatic scoliosis. Am. J. Surg..

[2] Kuznia A.L., Hernandez A.K., Lee L.U. (2020). Adolescent Idiopathic Scoliosis: Common Questions and Answers. Am. Fam. Physician..

[3] Xu S., Su Y., Wang Z., Liu C., Jin L., Liu H. (2021). Characteristics of scoliosis in primary and secondary school students in Chinese mainland: A meta-analysis of 72 studies. Chin. J. Spine Spinal Cord..

[4] Hong J.Y., Suh S.W., Park H.J., Kim Y.H., Park J.H., Park S.Y. (2011). Correlations of adolescent idiopathic scoliosis and pectus excavatum. J. Pediatr. Orthop..

[5] Basbug G., Gurses H.N., Zeren M., Elmadag N.M. (2023). Effects of inspiratory muscle training on respiratory muscle strength, respiratory function and functional capacity in adolescents with idiopathic scoliosis: A randomized, controlled trial. Wien Klin Wochenschr..

[6] Editorial Department of Journal of Capital University of Physical Education (2023). The concept and implementation of the integration of sports and medicine from the perspective of healthy China—A review of the first China Conference on Physical Education and Health. J. Capital. Univ. Phys. Educ..

[7] Wu Q., Zhang P., Chen J. (2021). Interpretation of the 2016 SOSORT guidelines: Orthopedic and rehabilitation treatment of idiopathic scoliosis. Reflexology Rehabil. Med..

[8] Fiore M., Ruffilli A., Viroli G., Barile F., Manzetti M., Faldini C. (2022). Minimally invasive surgery using posterior-only Pedicle screw fixation in treatment of Adolescent Idiopathic Scoliosis: A Systematic Review and Meta-Analysis. J. Clin. Neurosci..

[9] Sheffer B.W., Kelly D.M., Rhodes L.N., Sawyer J.R. (2017). Perioperative Pain Management in Pediatric Spine Surgery. Orthop. Clin. North. Am..

[10] Negrini S., Donzelli S., Aulisa A.G., Czaprowski D., Schreiber S., de Mauroy J.C., Diers H., Grivas T.B., Knott P., Kotwicki T. (2018). 2016 SOSORT guidelines: Orthopaedic and rehabilitation treatment of idiopathic scoliosis during growth. Scoliosis Spinal Disord..

[11] Negrini S., Minozzi S., Bettany-Saltikov J., Chockalingam N., Grivas T.B., Kotwicki T., Maruyama T., Romano M., Zaina F. (2010). Braces for Idi-Opathic Scoliosis in Adolescents.

[12] Fusco C., Zaina F., Atanasio S., Romano M., Negrini A., Negrini S. (2011). Physical exercises in the treatment of adolescent idiopathic scoliosis: An updated sys-tematic review. Physiother. Theory. Pract..

[13] Burger M., Coetzee W., du Plessis L.Z., Geldenhuys L., Joubert F., Myburgh E., van Rooyen C., Vermeulen N. (2019). The effectiveness of Schroth exercises in adolescents with idiopathic scoliosis: A systematic review and meta-analysis. South Afr. J. Physiother..

[14] Park J.-H., Jeon H.-S., Park H.-W. (2018). Effects of the Schroth exercise on idiopathic scoliosis: A meta-analysis. Eur. J. Phys. Rehabil. Med..

[15] Ceballos-Laita L., Carrasco-Uribarren A., Cabanillas-Barea S., Pérez-Guillén S., Pardos-Aguilella P., DEL Barrio S.J. (2023). The effectiveness of Schroth method in Cobb angle, quality of life and trunk rotation angle in adolescent idiopathic scoliosis: A systematic review and meta-analysis. Eur. J. Phys. Rehabil. Med..

[16] Page M.J., McKenzie J.E., Bossuyt P.M., Boutron I., Hoffmann T.C., Mulrow C.D., Shamseer L., Tetzlaff J.M., Akl E.A., Brennan S.E. (2021). The PRISMA 2020 statement: An updated guideline for reporting systematic reviews. BMJ.

[17] Higgins J.P.T., Altman D.G., Gøtzsche P.C., Jüni P., Moher D., Oxman A.D., Savović J., Schulz K.F., Weeks L., Sterne J.A.C. (2011). The cochrane collaboration’s tool for assessing risk of bias in randomised trials. BMJ.

[18] Schreiber S., Parent E.C., Moez E.K., Hedden D.M., Hill D., Moreau M.J., Lou E., Watkins E.M., Southon S.C. (2015). The effect of Schroth exercises added to the standard of care on the quality of life and muscle endurance in adolescents with idiopathic scoliosis-an assessor and statistician blinded randomized controlled trial: “SOSORT 2015 Award Winner”. Scoliosis.

[19] Shi J.H. (2022). Effect of Schroth training combined with orthosis in adolescent scoliosis. Chin. J. Pract. Med..

[20] Schreiber S., Parent E.C., Moez E.K., Hedden D.M., Hill D.L., Moreau M., Lou E., Watkins E.M., Southon S.C. (2016). Schroth Physiotherapeutic Scoliosis-Specific Exercises Added to the Standard of Care Lead to Better Cobb Angle Outcomes in Adolescents with Idiopathic Scoliosis—An Assessor and Statistician Blinded Randomized Controlled Trial. PLoS ONE.

[21] Kim G., HwangBo P.N. (2016). Effects of Schroth and Pilates exercises on the Cobb angle and weight distribution of patients with scoliosis. J. Phys. Ther. Sci..

[22] Kuru T., Yeldan I., Dereli E.E., Özdinçler A.R., Dikici F., Çolak I. (2016). The efficacy of three-dimensional Schroth exercises in adolescent idiopathic scoliosis: A randomised controlled clinical trial. Clin. Rehabil..

[23] Mohamed R.A., Yousef A.M. (2021). Impact of Schroth three-dimensional vs. proprioceptive neuromuscular facilitation techniques in adolescent idiopathic scoliosis: A randomized controlled study. Eur. Rev. Med. Pharmacol. Sci..

[24] Kocaman H., Bek N., Kaya M.H., Büyükturan B., Yetiş M., Büyükturan Ö. (2021). The effectiveness of two different exercise approaches in adolescent idiopathic scoliosis: A single-blind, randomized-controlled trial. PLoS ONE.

[25] Akyurek E., Zengin Alpozgen A., Akgul T. (2022). The preliminary results of physiotherapy scoliosis-specific exercises on spine joint position sense in adolescent idiopathic scoliosis: A randomized controlled trial. Prosthet. Orthot. Int..

[26] Zapata K.A., McIntosh A.L., Jo C.H., Virostek D. (2023). The Addition of Daytime Physiotherapeutic Scoliosis-specific Exercises to Adolescent Idiopathic Scoliosis Nighttime Bracing Reduces Curve Progression. J. Pediatr. Orthop..

[27] Moawd S.A., Nambi G., El-Bagalaty A.E., Hassan S.M., Elsayed S.E.B., Aboelmagd F.M., Alhwoaimel N.A., Abdeen H.A. (2023). Combined effect of Schroth method and Gensingen brace on Cobb’s angle and pulmonary functions in adolescent idiopathic scoliosis: A prospective, single blinded randomized controlled trial. Eur. Rev. Med. Pharmacol. Sci..

[28] Kurak K., Altunhan A., Açak M., Korkmaz M.F., Düz S. (2022). The Effect of Electrical Muscle Stimulation (EMS) Enhanced Schroth Method Training on Cobb Angle and Quality of Life in Patients with Scoliosis. Pak. J. Med. Health Sci..

[29] Li N., Wang L. (2021). Efficacy of Schrosss gymnastics in moderate adolescent idiopathic scoliosis. Chongqing Med..

[30] Xu R., Huang J. (2022). Effect of “Three Steps and Seven Methods” Massage in Adolescent Idiopathic Scoliosis. Chin. J. Contemp. Med..

[31] Lu Y., Luo G., Xie H., Dai Z. (2022). Effect of Schroth therapy combined with Chinese massage in the rehabilitation of adolescent idiopathic scoliosis. Chin. J. General. Pract..

[32] Kouwenhoven J.-W., Castelein R.M. (2008). The pathogenesis of adolescent idiopathic scoliosis: Review of the literature. Spine.

[33] Lau K.K.L., Law K.K.P., Kwan K.Y.H., Cheung J.P.Y., Cheung K.M.C. (2023). Proprioception-related gene mutations in relation to the aetiopathogenesis of idiopathic scoliosis: A scoping review. J. Orthop. Res..

[34] Cheng J.C., Castelein R.M., Chu W.C., Danielsson A.J., Dobbs M.B., Grivas T.B., Gurnett C.A., Luk K.D., Moreau A., Newton P.O. (2015). Adolescent idiopathic scoliosis. Nat. Rev. Dis. Primers.

[35] Lehnert-Schroth C. (1992). Introduction to the Three-Dimensional Scoliosis Treatment According to Schroth. Physiotherapy.

[36] Ying X.M., Lv L.J., Zhang H.Y., Pan Y.S., Li S.L., Li X.M., Ye X., Yang C., He L.L. (2023). Correlation between Cobb Angle and straight spinous process Angle in adolescent idiopathic scoliosis. China Orthop. Inj..

[37] Bezalel T., Carmeli E., Levi D., Kalichman L. (2019). The Effect of Schroth Therapy on Thoracic Kyphotic Curve and Quality of Life in Scheuermann’s Patients: A Randomized Controlled Trial. Asian Spine J..

[38] Pugacheva N. (2012). Corrective Exercises in Multimodality Therapy of Idiopathic Scoliosis in Children-Analysis of Six Weeks Efficiency Pilot Study. Stud. Health Technol. Inform..

[39] Wong C.K.H., Cheung P.W.H., Samartzis D., Luk K.D.-K., Cheung K.M.C., Lam C.L.K., Cheung J.P.Y. (2017). Mapping the SRS22r questionnaire onto the EQ-5D-5L utility score in patients with adolescent idiopathic scoliosis. PLoS ONE.

[40] Lowe T.G., Burwell R.G., Dangerfield P.H. (2004). Platelet calmodulin levels in adolescent idiopathic scoliosis (AIS): Can they predict curve progression and severity? Summary of an electronic focus group debate of the IBSE. Eur. Spine J..

[41] Asher M., Lai S.M., Burton D., Manna B. (2003). Discrimination validity of Scoliosis Research Society-22 patient questionnaire: Relation-ship to idiopathic scoliosis curve pattern and curve size. Spine.

[42] Li K.R., Zhao Z., Chen M.X. (2023). Pulmonary function and respiratory muscle strength in adolescent patients with mild to moderate idiopathic scoliosis. J. Kunming Med. Univ..

